# The Influence of Cup Orientation on the Primary Fixation of a Hemispherical Cementless Acetabular Cup: A Cohort Based Finite Element Study

**DOI:** 10.1002/jor.26084

**Published:** 2025-04-12

**Authors:** Mark Taylor, Stuart Callary, Dominic Thewlis, Rebecca Bryan

**Affiliations:** ^1^ Medical Device Research Institute, College of Science and Engineering Flinders University Tonsley South Australia Australia; ^2^ Centre for Orthopaedic and Trauma Research, The Adelaide Medical School University of Adelaide Adelaide South Australia Australia; ^3^ Department of Orthopaedics and Trauma Royal Adelaide Hospital Adelaide South Australia Australia; ^4^ Synopsys Northern Europe ltd Exeter UK

**Keywords:** arthroplasty‐hip, biomechanics, finite element analysis, implant fixation

## Abstract

Cup orientation has been investigated in detail with respect to risk of dislocation, however, the impact on the primary fixation of cementless cups is poorly understood. The aim of this study was to assess the influence acetabular component orientation on the primary fixation of cementless acetabular cups using an in silico clinical trial framework. Finite element models of 57 implanted hemi‐pelves were generated from CT scans of a cohort of end stage osteoarthritis patients. Each hemi‐pelvis was implanted with the with cup orientations that bounded the Lewinnek safe zone (mechanical alignment +/−10 degrees) and an approximation of the extreme orientations reported in the literature (mechanical alignment +/−20 degrees). Bone strain immediately adjacent to the implant and micromotions (gap and shear micromotions) were used to assess primary fixation. Analysis was performed at the levels of the individual subjects and the entire cohort. There was minimal variation in all metrics within the Lewinnek safe zone. Micromotion, particularly inferior gaping, was more sensitive to cup orientation than peri‐prosthetic bone strain, tending to increase with inclination angle. Both the peri‐prosthetic bone strains and micromotions were moderately correlated to the average bone modulus. Individuals with low bone modulus were shown to be more sensitive to changes in cup orientation for both peri‐prosthetic bone strains and micromotions both within and outside the Lewinnek safe zone. This suggests that assessing bone quality should be routinely incorporated into the planning process, particularly when considering cup orientations outside of the Lewinnek safe zone.

## 1 Introduction

Cementless acetabular cups are widely used in primary joint replacement, accounting for between 76% [1] and 95% [2] of preferred implant usage, either as part of a fully cementless or hybrid total hip replacement. Aseptic loosening accounts for approximately 20% of all revision surgeries in Australia [2]. Achieving primary fixation of cementless acetabular components is critical for ensuring longevity of the device. The initial mechanical environment, particularly the peri‐prosthetic bone strains and the associated micromotions at the bone implant interface are dependent on a number of factors including cup placement and orientation [3, 4, 5, 6, 7, 8]. Mechanical alignment of the acetabular component, namely 40 degrees inclination and 15 degrees of version, is still a widely used intraoperative target for acetabular cup placement [9]. To improve functional range of motion and reduce the incidence of impingement and dislocation, alternative alignment strategies have been explored, including anatomical and kinematic alignment [9]. These methods adjust the inclination and/or version of the cup to account for patient anatomy. Despite surgeon intentions, significant variation in the cup position achieved within patient cohorts continue to be reported in the literature. Esposito et al. reported acetabular cup inclinations ranging from 21 to 58 degrees and version from 2 to 40 degrees [10]. Based on analysis of planar X‐rays, Di Maro et al. reported a wider range of inclination and version from 18 to 69 degrees and 5 to 59 degrees, respectively [11].

Historically, acetabular cups have been oriented free hand at the time of surgery. Saxler et al [12] assessed the accuracy of free hand cup placement in a cohort of 103 hips using CT and reported inclination angles from 23 to 71 degrees (mean = 45.8, STD = 10.1 degrees) and version angles from −23 to 59 degrees (mean 27.3, STD = 15 degrees). It should be noted only three hips were reported to be retroverted. Patient specific guided instrumentation and navigation systems have been developed in an attempt to improve cup placement. Using patient specific instrumentation, Spencer Gardner [13] reported a wide variation, from 29 to 58 degrees inclination and 10 to 36 degrees of version, although the mean absolute deviation from the planned orientation was 3.9 degrees (range 0 to 13.6 degrees) and 3.6 degrees (range 0 to 12.9 degrees) for inclination and version respectively. Naito et al [14] reported on the use of an imageless navigation system with a target of 40 degrees of inclination and 20 degrees of anteversion and reported inclination and version angles with respect to the functional pelvic plane (FPP) ranging from 25 to 55 degrees and 0 to 34 degrees respectively. Regardless of the intended surgical plan (mechanical, anatomic or kinematic alignment), the use of patient specific instrumentation or navigation, there remains a wide range in the achieved cup orientation and the impact on the primary stability of cementless acetabular cups is unclear.

Finite element analysis has been used extensively to investigate the initial mechanical environment of cementless acetabular cups. Studies have consistently highlighted that the magnitude of interference [5, 15, 16, 17] and bone properties [5, 16, 18] affect the initial mechanical environment. Increasing interference is associated with greater interfacial bone strains and lower micromotions [15, 16]. Intra‐subject variability may be influenced by differences in bone morphology and bone properties. Lower bone modulus has been shown to increase interfacial bone strain and micromotions [5, 18]. A limitation of the majority of FE studies is that they only model a single representative individual [16, 19, 20, 21, 22, 23] or a small group of individuals [7, 24]. As far as the authors are aware, only O'Rourke et al [6] has applied FE to a large cohort and reported significant variation in bone‐implant micromotions, largely due to the individual variation in acetabular bone properties. To date, there has been minimal work to assess its influence on the primary fixation of cementless acetabular components. Clarke et al [7] examined the influence of patient and surgical factors, including cup orientation, on the bone‐implant micromotion of a press‐fit resurfacing acetabular component. Three cup inclination angles (35.7, 45.8 and 55.8) and three version angles (12.3, 27.3 and 42.3) were examined in a full factorial study on four different subjects, in addition to the degree of interference and the depth of insertion. In comparison with the other factors, cup orientation appeared to have minimal effect on predicted micromotion. Nie et al. [4] examined the effect of both cup orientation (combinations of inclinations angles from 35 to 50 degrees and version angles from 10 to 25 degrees) and superior cup placement (from 0 to 15 mm). Simulations were performed using a bonded bone‐implant interface and superior cup position was found to have a greater influence on micromotion than cup orientation. These studies suggest that cup orientation has a limited impact of the initial mechanical environment. However, both Clarke et al. and Nie et al only explored cup orientations within or close to the Lewinnek safe zone (40 degrees inclination, 15 degrees version +/−10 degrees) [25] and these studies were performed on a limited number of individuals, four and two hemi‐pelves, respectively. So, while we have some understanding of the initial mechanical environment in ideally orientated cups within the Lewinnek safe zone (LSZ), to date, there have been no studies that have explored the impact of the wider range of cup orientations reported in the literature.

The aim of this study was to investigate the influence of cup orientation on the primary fixation of cementless acetabular prostheses. A secondary aim was to assess the contribution of bone properties on the effect on primary fixation in a range of different cup orientations.

## 2 Methods

Finite element models of 57 implanted hemi‐pelvii were generated from CT scans using a custom scripted work‐flow (Simpleware™ software, Version 2022 T; Synopsys Inc., Sunnyvale, USA). The models were generated from a cohort of end‐stage osteoarthritis patients awaiting total hip replacement surgery. The cohort consisted of 22 individuals receiving their first hip replacement, 2 individuals who went on to receive bi‐lateral primary hip replacements and 9 individuals who had already received one hip replacement and were having the contralateral hip replaced. In the first hip replacement group, 44 models were generated using both sides of the pelvis. Analysis of the OA and contralateral sides showed that there was a mean absolute difference in the global average modulus of 72 MPa (range 0 to 214 MPa) and a mean increase in 1 cup size (range 0 to 3 cup sizes). Due to these differences in modulus and cup size, for the purpose of this study each hemi‐pelvis in this group were treated as unique.

The CT scans were auto‐segmented to identify the left and right hemi‐pelvis (Simpleware™ software, Version 2022 T; Synopsys Inc., Sunnyvale, USA). An anatomical reference frame was defined using the anterior superior iliac spines and symphysis pubis tubercle and the CT scans and segmented pelvii re‐orientated with respect to the anterior pelvic plane. Right hemi‐pelvii were mirrored, so that all analyses were performed on a left hemi‐pelvis. The centre of the left acetabulum was calculated using a least squares sphere fit and used as an initial estimate of the position of the acetabular cup. Each acetabulum was then fitted with idealized titanium alloy (*E* = 110 GPa) hemi‐spherical acetabular cups of increasing size from 44 to 68 mm in diameter in 2 mm increments, to represent the size range of commercial systems. The median cup size was 56 mm and ranged from 46 to 68 mm. The cups were mechanically aligned, in 40 degrees of inclination and 15 degrees of anteversion. The optimal cup size was determined by minimizing the sum of the gaps between the bone surface and the acetabular cup. The final position of the cup was refined by moving it in the AP, ML and IS directions and again minimising the sum of the gaps between the bone surface and the acetabular cup. A Boolean operation was then performed to prepare the cavity for the implant. The resulting models were meshed with 1st order tetrahedral elements. The global element edge length was set to 3 mm and a local element edge length of 0.75 mm was defined within a 5 mm layer of the acetabular shell. The local element edge length was selected based on previous research that has shown that convergence only occurs when the finite element edge length is similar to the CT image voxel resolution [26]. This resulted in bone meshes having between 230,000 and 457,000 elements and the total number of elements in the models ranging from 320,000 to 827,000 elements.

**Figure 1:**
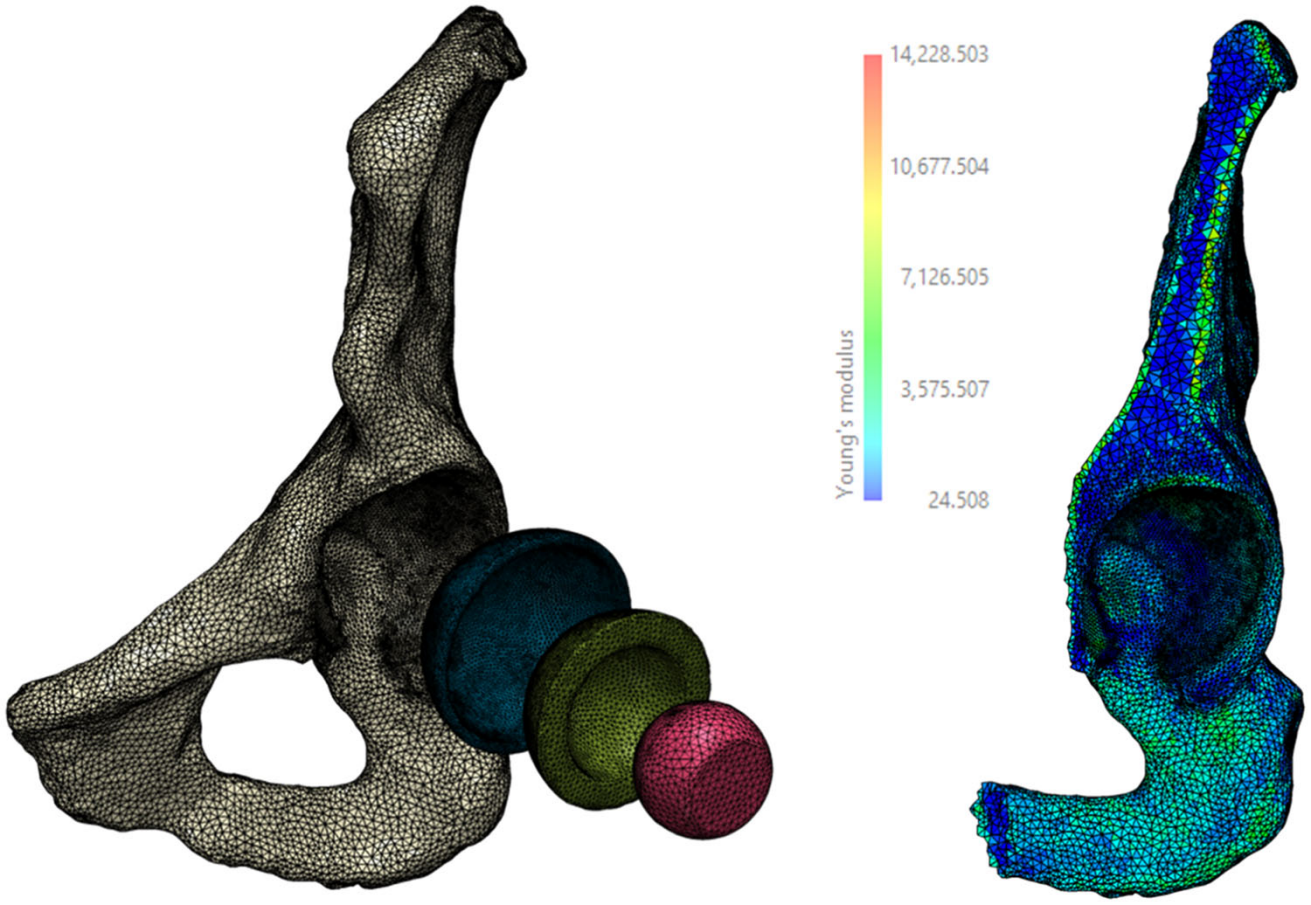
Exploded view of a representative finite element model of the implanted hemi‐pelvis (left) and cross section showing the assigned heterogenous bone modulus properties (right).

Each CT scan was calibrated using an air, fat and muscle calibration [27] and inhomogeneous material properties applied to the bone [28]. The cortex was represented using a 1.5 mm thick layer of shell elements (*E* = 17 GPa) over the entire hemi‐pelvis except the reamed portion of the acetabulum [29]. The hemi‐pelvis was rigidly fixed at the sacro‐iliac joint and the pubic symphysis [30]. The hip joint contact forces associated with level walking gait were discretised into 8 separate load cases (as per Dalstra et al [29]) and were applied through a 32 mm CoCr femoral head (*E *= 200 GPa) and a polyethylene liner (*E* = 0.8 GPa) (Figure 1). A Poisson's ratio of 0.3 was assumed for all materials except the polyethylene which was assumed to be 0.45. The joint contact force was scaled based on the size of the of acetabular component, simulating body weights from 500 to 1100 N for implant diameters of 44 to 68 mm, respectively. Muscle forces were ignored and this assumption has been shown to have negligible effect on the predicted peri‐prosthetic strains and micromotions [31]. Frictional contact (*μ* = 0.5) was modelled between the acetabular shell and the acetabular bone [32], but no interference was modelled. The median and 95th percentile of the composite peak strain (CPS) was reported for the bone within a 2 mm radius of the acetabular shell [24] as a measure of the risk of bone failure and a surrogate measure of the risk of implant migration [24]. The CPS captures the peak strain at each individual node throughout the gait cycle. The median and 95th percentile of the composite peak gap (CPG) and composite peak shear micromotion (CPSM) at the bone implant‐interface were also predicted as surrogate measures of the potential for osseointegration [5, 6, 24]. All analyses were performed using Ansys 2022 R1 (Ansys, Canonsburg, USA).

Mechanical alignment (40 degrees/15 degrees inclination/anteversion) was assumed to be the reference orientation. For each individual, the same sized cup was also implanted in the following orientations: 30 degrees/5 degrees; 30 degrees/25 degrees; 50 degrees/5 degrees; 50 degrees/25 degrees; 20 degrees/−5 degrees; 20 degrees/35 degrees; 60 degrees/−5 degrees and 60 degrees/35 degrees. Effectively, this represents the bounding box of the so called Lewinnek safe zone (40 degrees/15 degrees +/−10 degrees) and a second bounding box that approximates the extremes of cup placement reported in the literature (40 degrees/15 degrees +/−20 degrees).

### 2.1 Analysis and Statistics

Linear regression analysis was performed on the median and 95th percentile CPS, CPG and CPSM against the average global bone modulus and cup size for the baseline cup orientation.

Analysis of the data was performed both at the cohort level and at that of the individuals. To gain an understanding of the influence of cup orientation, surface response plots were generated for the cohort averaged median and 95th percentile of the CPS, CPG and CPSM. The sensitivity of an individual to implant orientation was also assessed. For each individual within the cohort, the change (the difference between the maximum and minimum values) across the five unique implant orientations within the LSZ and for all nine implant orientations was calculated for the median and 95th percentile of the CPS, CPG and CPSM. Regression analysis was performed on these metrics with respect to the average bone modulus. In addition, categorical analysis was performed, by comparing these metrics across three hemi‐pelvis averaged bone modulus groups, low (less than 750 MPa, *N* = 15), intermediate (between 750 and 1000 MPa, *N* = 26) and high average modulus (above 1000 MPa, *N* = 15).

## 3 Results

There was approximately a fivefold variation in the predicted median of the CPS (range from 1000 to 5200 microstrain) of the baseline mechanically aligned cup across the cohort. Both the median and 95th percentile of the CPS were moderately correlated to the mean pelvis modulus with coefficient of determination of 0.577 and 0.63 respectively.

**Figure 2:**
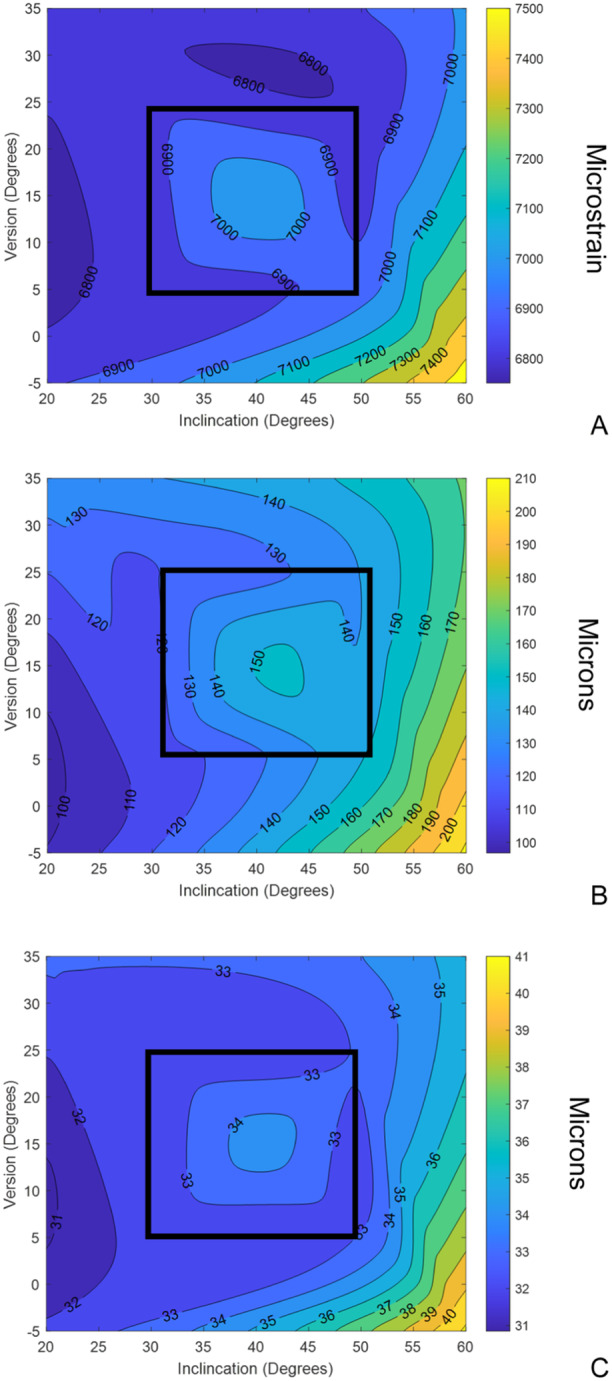
Response surface of the cohort averaged: (A) 95th percentile CPS, (B) 95th percentile CPG and (C) 95th percentile CPSM due to variation in the inclination and version angle. The Lewinnek safe zone is marked by the black box.

**Figure 3:**
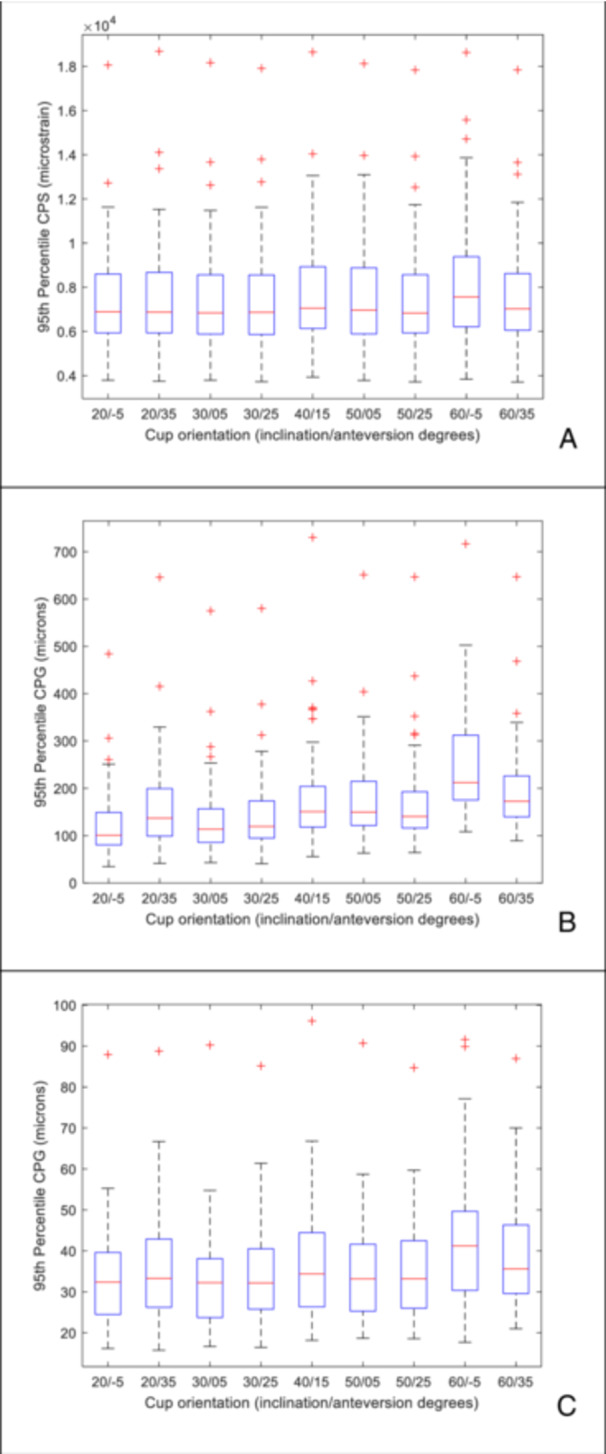
Box plot of the 95th percentile (A) CPS, (B) CPG and (C) CPSM for the cohort at each implant orientation. Red line is the median, lower bound of box is the 25th percentile, the upper bound of the box is the 75th percentile. The whiskers are the maximum and minimum values and the crosses are outliers.

**Figure 4:**
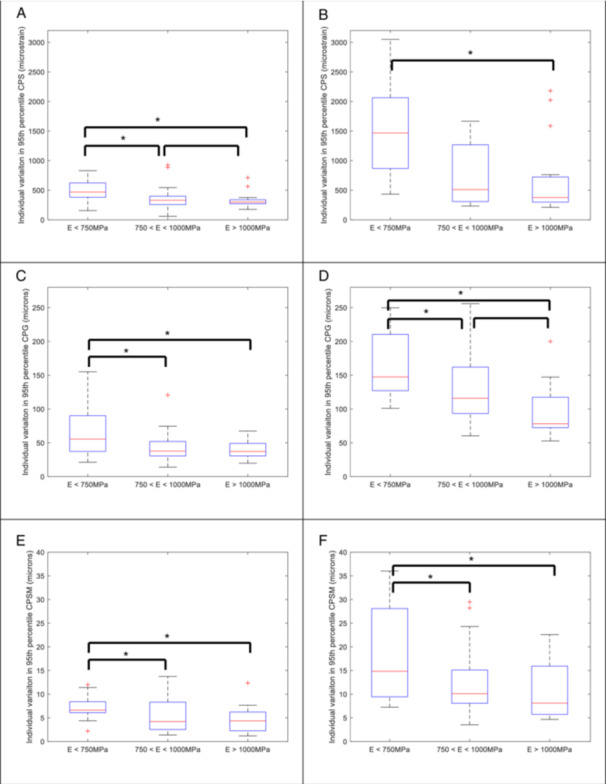
Categorical analysis of the individual variation in the 95th percentile of the CPS (A, B), CPG (C, D) and CPSM (E, F) within the LSZ (A, C, E) and across all acetabular cup orientations (B, D, F) as a function of the average bone modulus. Red line is the median, lower bound of box is the 25th percentile, the upper bound of the box is the 75th percentile. The whiskers are the maximum and minimum values and the crosses are outliers. *indicates statistically significant difference (*p* < 0.05).

As similar trends were observed in the median (Appendix, figures A1‐3) and the 95th percentile of the CPS data, only the latter will be described in detail. There was minimal variation in the cohort averaged 95th percentile of the CPS (Figure 2A) within the LSZ of cup alignment (range 6840 to 7050 microstrain). Outside of the LSZ, there was an increase in the 95th percentile of the CPS as the acetabular cup inclination increased to 60 degrees and was also retroverted by 5 degrees, with a maximum cohort averaged 95th percentile of the CPS of 7560 microstrain. Similar distributions across the cohort of the 95th percentile of the CPS were seen at all implant orientations (Figure 3A), except at 60/−5 degrees (inclination/version) which resulted in elevated cohort averaged CPS, but also greater variability. At the individual subject level, again, across the majority of the cohort there was minimal change in the CPS, with the cohort averaged change in CPS being 605 microstrain, as cup alignment varied. However, there were individuals that had up to 3050 microstrain change in the 95th percentile of the CPS as the cup orientation was altered. There was no correlation between the change in 95th percentile of the CPS and the average bone modulus. Categorical analysis within (Figure 4A) and outside the LSZ (Figure 4B) shows that changes in the 95th percentile of the CPS due to changes in cup orientation were greater in the low modulus group as compared to the intermediate and high modulus groups. Although lower in magnitude, statistically significant differences were seen between the low and intermediate (*p* = 0.033), the low and high (*p* = 0.007) and the intermediate and high modulus groups (*p* = 0.038) within the LSZ. Outside of the LSZ the individual variation in strain was much greater with statistically significant differences seen between the low and intermediate (*p* = 0.0004) and the low and high modulus groups (*p* = 0.0059).

On loading, due to the superiorly directed load, a gap tends to open on the inferior aspect of the cup as has been previously reported in the literature [33]. Again, as similar trends were seen for the median (Appendix, figures A1‐3) and 95th percentile data, only the later will be described in detail. Across the cohort for the baseline, mechanically aligned cup, the 95th percentile of the CPG varied from 55 to 730 microns. There was a weak correlation between the 95th percentile of the CPG and the average bone modulus and acetabular cup size of 0.48 and 0.49, respectively.

There was minimal variation in the cohort averaged 95th percentile of the CPG within the Lewinnik safe zone, with the cohort averaged 95th percentile of the CPG varying from 140 to 184 microns (Figure 2B). Outside of the Lewinnek safe zone, as the cup inclination increased and version decreased, there was an increase in the cohort averaged 95th percentile of the CPG to a maximum of 252 microns. At cup inclinations below 30 degrees, independent of the version angle, the cohort averaged 95th percentile of the CPG remained below 150 microns. Examining the behaviour across the entire cohort (Figure 3B), there was a general trend that as the inclination angle increased, not only did the magnitude of the 95th percentile of the CPG increase, but so did the degree of variation, particularly at 60 degrees of inclination. At the individual subject level altering the cup alignment resulted in substantial differences in the magnitude of the gap, with the cohort averaged change in 95th percentile CPG of 130 microns and a maximum change seen with an individual of 255 microns. There was no correlation between individual variation of CPG and bone modulus, however categorical analysis shows that the degree of change in CPG was greater in lower average bone modulus group (Figure 4C and D), with significant differences seen between all modulus groups both within the LSZ (except the intermediate to high bone modulus groups), albeit it at lower magnitude, and outside of the LSZ.

In comparison with CPG, the CPSM magnitudes are lower. For the baseline, mechanically aligned acetabular cup, the cohort averaged median of the CPSM was 8 microns (range from 1 to 19 micron) and cohort averaged 95th percentile of the CPSM was 34.3 microns (individuals range from 18 to 96 microns). There was a moderate correlation (*R* ^2^ = 0.74) for the median and a weak correlation (*R* ^2^ = 0.38) 95th percentile of the CPSM with average modulus. There was no correlation of the CPSM metrics with cup size. There is only minor variation in the cohort averaged 95th percentile (range from 33 to 42 microns) across the full range of implant orientations. At the individual level, the change in 95th percentile of the CPSM was less than 40 microns. There was no correlation between the change in CPSM and the bone modulus, however, the categorical analysis (Figure 4E and F) again showed that there was a statistically significant increased variation within (low modulus vs high modulus (*p* = 0.022)) and outside of the LSZ (low modulus bone group as compared to the intermediate (*p* = 0.036) and high modulus groups (*p* = 0.0186)).

## 4 Discussion

Various alignment strategies and delivery methods have developed to extend the functional range of motion and to reduce the risk of impingement and dislocation [9]. Mechanical alignment is widely adopted, however, there is debate as to whether the Lewinnek safe zone is an appropriate target to minimise the risk of dislocation [34]. Regardless of the choice of alignment strategy or delivery method, there is significant variation in the achieved inclination and version angle of the acetabular cup reported in the literature. The potential implications on the primary fixation of cementless acetabular cups, in terms of the peri‐prosthetic bone strains and micromotion, both within and outside the Lewinnek safe zone are to date poorly understood.

At the cohort level, acetabular cup orientation had minimal effect on the periprosthetic cup strains, interfacial gap opening and shear micromotions within the Lewinnek safe zone (40/15 degrees +/−10 degrees). This suggests that achieving mechanical alignment will produce a consistent initial mechanical environment. Outside of the Lewinnek safe zone, cup orientation had the greatest effect on the interfacial gap. Above 50 degrees of inclination, the cohort averaged 95th percentile of the interfacial gap increased from approximately 140 microns to greater than 180 microns depending on the version angle. Although there is an increase in the inferior bone‐implant interface gap as the inclination angle increases, this did not appear to result in similar magnitudes of increase in either the periprosthetic bone strains or the shear micromotions. All metrics reached their maximal values with a cup inclination of 60 degrees and retroverted 5 degrees. Although such an extreme position is unlikely to be intended, clinical studies have reported that these cup orientations can and do occur [12].

Across the cohort there was considerable variation in all of the metrics with approximately a fivefold variation in the peri‐prosthetic strains, a 10 fold variation in the interfacial gap and a fivefold variation in the shear micromotions. This is likely to be due variation in patient related factors including changes in morphology and bone properties across the cohort [5, 6]. Weak to moderate correlations were found for the CPS and CPG and bone modulus, with individuals with low mean modulus tending to experience higher peri‐prosthetic strains and larger interfacial gap opening. The range in the interfacial gaps and shear micromotions are similar to those previously reported by O'Rourke et al. [6] who reported a median 95th percentile composite peak resultant micromotion of 136 microns, with a range of 18 to 624 microns, in a cohort of acetabular cups implanted in healthy hemi‐pelvii.

For the peri‐prosthetic bone strains, the minor variation in the cohort averages and the similar degree of variation at each cup position would suggest that the changes in cup orientation are insensitive to patient related factors both within and outside the Lewinnek safe zone. The interfacial gap and to a lesser extent the shear micromotions, tend to increase with inclination angle and the degree of variation was similar for the majority of cup positions, suggesting that within the Lewinnek safe zone the changes in gap and shear micromotions due to cup position are not influenced by patient related factors. The exception is at the extreme position of 60 degrees of inclination and ‐5 degrees of version, where there is not only a substantive increase in the cohort average, but also in the degree of variability across the group. This suggests that in this position, the interfacial gap becomes more sensitive to patient related factors. However, only looking at the behaviour at a cohort level is an over simplification and potentially hides important trends. An advantage of using a cohort based FE study is the ability to examine all cup orientations within each subject and to assess the impact at the individual level. Changes in cup orientation resulted in variations in the 95th percentile of the CPs from 200 to 3000 microstrain, in the CPG from 60 to 260 microns and in the CPSM from 5 to 35 microns. There was no correlation between an individual's change in any of these variables and the average pelvis modulus. However, the categorical analysis based on the average pelvis modulus clearly showed that the low modulus group (*E* < 750 MPa) had statistically significantly greater variability than the medium and high bone modulus groups for the periprosthetic strains, interfacial gap and shear micromotions. This was observed within the LSZ but was amplified outside of the LSZ (Figure 4). This implies that individuals with low bone modulus are more sensitive to cup mal‐orientations and navigational aids should be used to ensure that the desired cup placement is achieved. This coupled with the fact that both the peri‐prosthetic strains and interfacial gaps are greater in low modulus bone suggests that assessing bone quality should be routinely incorporated into the planning process. In the absence of quantitative bone assessment, a conservative plan to stay within the Lewinnek safe zone would be recommended. Conversely, the comparative small variation observed in the high modulus group suggests that this group is robust to variation in cup placement, allowing for greater latitude outside of the Lewinnek safe zone when planning appropriate cup placement to minimise the risk of dislocation.

There are a number of limitations to the findings of our study. The joint contact forces were simply scaled as a function of the acetabular cup size. As the main focus of the study was to explore the influence of cup orientation, as such each hemi‐pelvis acted as its own comparator and thus this assumption does not affect the findings of this study. No interference was modelled at the implant‐bone interface. The nominal radial interference is typically 1 mm, but the true interference achieved during surgery is poorly understood. The size of the reamed cavity has been reported to be 0.5 to 1 mm larger than intended [35, 36] resulting in a lower interference than planned. This is further complicated by the viscoelastic properties of the bone that lead to significant stress relaxation in the immediate post operative period [36, 37]. In the absence of a detailed understanding of the true interference, a worst case of no interference was assumed. Categorical analysis has highlighted that low bone modulus group is more susceptible to changes in cup alignment. This may warrant further study using a more systematic approach to fully understand the influence of bone properties in conjunction with cup alignment. Finally, this is purely a simulation based study and there has been no validation or corroboration with experimental data. Although validation versus in vitro data is important, with the development of in silico clinical trials and implementation of simulation into surgical planning, there is a pressing need to validate finite element models with clinically measurable outcomes, such as implant migration measured using Radiostereometric Analysis (RSA). Recently a corroboration between FE predictions and early migration of cementless acetabular cups has been reported [24]. Using a similar model generation methodology to this study, including no inclusion of an interfence fit, Fallahnehzad et al [24] reported that the FE predicted peri‐prosthetic strain was moderately correlated (*R* ^2^ = 0.69) to in vivo implant migration measured by RSA at 3 months. Categorical analysis showed that predicted micromotions followed a similar trend to the migration measured at 3 months. Similar to the current study, the individuals with low bone modulus were predicted to have elevated periprosthetic bone strains and bone‐implant micromotions.

In conclusion, there was minimal variation in both the peri‐prosthetic bone strains and interfacial micromotions within the Lewinnek safe zone. The interfacial gap was most sensitive to acetabular component orientation, tending to increase with an increase in inclination angle. The predicted peri‐prosthetic bone strains and micromotions were more sensitive to changes in cup orientation in low modulus bone. This suggests that screening for low bone density may assist the planning process and ensure a suitable target cup orientation for those identified.

## Author Contributions

**Mark Taylor:** conceptualisation, methodology, investigation, formal analysis, writing – original draft. **Stuart Callary:** data collection, writing – original draft. **Dominic Thewlis:** data collection, writing – original draft. **Rebecca Bryan:** conceptualisation, methodology, writing – original draft.

## Supporting information

JOR‐24‐0630_appendix.
