# Supplementary material for: The Influence of Cup Orientation on the Primary Fixation of a Hemispherical Cementless Acetabular Cup: A Cohort Based Finite Element Study
Source: J Orthop Res. 2025 Apr 12;43(7):1293–302. doi: 10.1002/jor.26084 (PMC12159585; doi:10.1002/jor.26084)
Supplement: Supplementary file 1 — JOR‐24‐0630_appendix. [file JOR-43-1293-s001.docx]

| A  Microstrain |
| --- |
| B  Microns |
| C  Microns |
| Figure A1 – Response surface of the cohort averaged: a) 50^th^ percentile CPS, b) 50^th^ percentile CPG and c) 50^th^ percentile CPSM due to variation in the inclination and version angle. The Lewinnek safe zone is marked by the black box. |
| A |
| B |
| C |
| Figure A2 – Box plot of the 50^th^ percentile a) CPS, b) CPG and c) CPSM for the cohort at each implant orientation. Red line is the median, lower bound of box is the 25^th^ percentile, the upper bound of the box is the 75^th^ percentile. The whiskers are the maximum and minimum values and the crosses are outliers |
| A |
| B |
| C |
| Figure A3 – Categorical analysis of the individual variation in the 95^th^ percentile a) CPS, b) CPG and c) CPSM across all acetabular cup orientations as a function of the average bone modulus. Red line is the median, lower bound of box is the 25^th^ percentile, the upper bound of the box is the 75^th^ percentile. The whiskers are the maximum and minimum values and the crosses are outliers. |
